# Risk Factors and Mortality in Elderly ARDS COVID-19 Compared to Patients without COVID-19

**DOI:** 10.3390/jcm11175180

**Published:** 2022-09-01

**Authors:** Davide Chiumello, Leo Modafferi, Isabella Fratti

**Affiliations:** 1 Department of Anesthesia and Intensive Care, ASST Santi Paolo e Carlo, San Paolo University Hospital, Via Di Rudini 9, 20142 Milan, Italy; 2 Department of Health Sciences, University of Milan, 20122 Milan, Italy; 3 Coordinated Research Center on Respiratory Failure, University of Milan, 20122 Milan, Italy

During the last few decades, due to the increase in elderly patients among the general population, the number of patients aged over 80 years admitted in intensive care significantly incremented [1]. Bagshaw et al. recently showed that elderly patients represented one in every four admissions to intensive care [2]. Pneumonia and sepsis have been reported as the two most common risk factors for acute respiratory distress syndrome (ARDS) [3].

The current incidence and mortality for ARDS are quite variable among countries. For example, in the United States, the incidence of ARDS was significantly higher compared to Europe, where it was found to reach up to 58 cases per 100.000 persons-years [3]. Moreover, the incidence increased with age, with up to 306 cases per 100.000 person-years for patients aged between 75 and 84 years [3].

Concerning critically ill patients with community pneumonia, an age over 80 years was an independent risk factor for death, both at 30 days (OR = 2.54 (1.2–5.3)) and at 1 year (OR = 3.47 (1.9–6.0)) [4]. Similarly, the mortality in ARDS increases up to 60% for patients up to 85 years, with a hazard ratio of 2.5 for hospital deaths [3,5]. Although previous data reported that after intensive care admission, young and older patients received different intensities of treatment in terms of mechanical ventilation, vasopressor infusion, and renal replacement therapy, more recently, studies found that the number of treatments for patients aged 80 years has increased [1,6,7].

In addition, elderly patients with ARDS presented a higher duration of mechanical ventilation and length of stay in intensive care [5]. The prediction of mechanical ventilation duration could affect both clinical management and intensive care resource utilization. Additionally, comorbidities, such as the presence of chronic respiratory disease, heart failure, cerebrovascular diseases, malnutrition, and end-stage renal failure, affected the duration of mechanical ventilation and the final outcome [8,9,10]. Unfortunately, an accurate prediction of mechanical ventilation is quite difficult for critical care physicians according to clinical data at admission. A possible solution may be the application of a machine learning technique according to the clinical features of ARDS evaluated in the first days of intensive care admission [11].

Since the first cases of coronavirus disease 2019 (COVID-19) reported in Wuhan, China, more than 416 million cases with 5.8 million deaths have been reported around the world [12,13]. Typically, COVID-19 can range from an asymptomatic infection to a severe form of ARDS. Unfortunately, other than vaccination as the most useful preventive measure, there are only a few available therapeutic treatments. Among these, invasive and noninvasive mechanical ventilation remain the most powerful symptomatic treatment [14].

Based on the first report in hospitalized patients from China, age was associated with death, similar to previous data from SARS and MERS infection [15,16,17,18]. Elderly patients, in addition to presenting more chronic diseases, could have a lower efficacy to respond to virus infection. In the largest cohort of hospitalized patients with COVID-19, the increased age was an independent risk factor for mortality, with odds risk approximately doubling for an age between 65 and 79 years compared to 50 and 64 years [19]. A systematic review showed that mortality from COVID-19 was independently associated with the patient’s age and older patients were more likely to be admitted to intensive care and develop ARDS [20]. Patients aged 70 years represented up to 28 of the total COVID-19 critically ill patients admitted to intensive care [21]. The factors independently associated with intensive care admission included age, the presence of immunosuppression, and diabetes [22].

The mortality for elderly patients admitted to intensive care ranged between 75 and 85%, significantly higher compared to that previously reported in non-COVID-19 patients [23,24,25]. Comparing ARDS in COVID-19 and not COVID-19, it was shown that COVID-19 patients presented a higher lung gas volume with lower extension of lung disease but a higher impairment in oxygenation [26]. By analyzing two cohorts of patients older than 80 years, it was found that COVID-19 patients presented a slightly lower age and SOFA, while being more likely to receive invasive mechanical ventilation and had a worse outcome, 38 vs. 57% [27]. Furthermore, there were no differences in the use of vasoactive drugs or renal replacement therapy. Regarding the hospital mortality, it was associated with age, race, immunosuppression, renal disease, neurologic disorders, and diabetes. The presence of at least three of these medical conditions was associated with a higher risk for intensive care admission and death [22].

In two studies that compared the outcomes, COVID-19 had significantly higher lethality compared to patients with influenza [28,29]. In particular, the mortality was 67% in patients aged 70–89 years with a severe case of COVID-19, compared to 45% in the influenza group for similar ages [28].

These data suggest that ARDS, due to COVID-19, in addition to age, comorbidities, the severity of infection, the disease progression, and inflammatory trajectory, could lead to a worse outcome compared to patients without COVID-19.
